# SIRPα-Fc fusion protein IMM01 exhibits dual anti-tumor activities by targeting CD47/SIRPα signal pathway via blocking the “don’t eat me” signal and activating the “eat me” signal

**DOI:** 10.1186/s13045-022-01385-2

**Published:** 2022-11-16

**Authors:** Jifeng Yu, Song Li, Dianze Chen, Dandan Liu, Huiqin Guo, Chunmei Yang, Wei Zhang, Li Zhang, Gui Zhao, Xiaoping Tu, Liang Peng, Sijin Liu, Xing Bai, Yongping Song, Zhongxing Jiang, Ruliang Zhang, Wenzhi Tian

**Affiliations:** 1 Department of Hematology, The First Affiliated Hospital of Zhengzhou University, Zhengzhou, 450052 Henan China; 2 ImmuneOnco Biopharmaceuticals (Shanghai) Co., Ltd., Shanghai, 201203 China

**Keywords:** CD47, Signal regulatory protein α (SIRPα), Immune checkpoint pathway, SIRPα-Fc fusion proteins, Cancer immunotherapy

## Abstract

A novel recombinant SIRPα-Fc fusion protein, IMM01, was constructed and produced using an in-house developed CHO-K1 cell expression system, and the anti-tumor mechanism of IMM01 targeting the CD47-SIRPα pathway was explored. The phagocytosis and in vitro anti-tumor activity of IMM01 were evaluated by antibody-dependent cellular phagocytosis (ADCP), antibody-dependent cell-mediated cytotoxicity (ADCC), and complement-dependent cytotoxicity (CDC) assays. In vivo mouse tumor model studies were used to explore therapeutic efficacy as well as the mechanism of action. An in vitro binding assay revealed that IMM01 has a strong binding affinity to CD47 with an EC50 of 0.4967 nM. IMM01 can induce strong ADCP and moderate ADCC, but not CDC. IMM01-induced strong phagocytosis against tumor cells was attributed to dual activities of blocking the "don’t eat me" signal and activating the "eat me" signal, and IMM01 exhibits strong and robust in vivo anti-tumor activities either as monotherapy on hematological malignancies, or in combination therapy with PD-L1 monoclonal antibody (mAb), PD-1 mAb, and HER-2 mAb on solid tumors. Finally, IMM01 demonstrated a favorable safety profile with no human RBC binding activity or hemagglutination induction. IMM01 inhibits the growth of tumor cells by the following three possible mechanisms: (1) directly activating macrophages to phagocytize tumor cells; (2) activated macrophages degrade phagocytized tumor cells and present tumor antigens to T cells through MHC molecules to activate T cells; (3) activated macrophages can convert "cold tumors" into "hot tumors" and increase the infiltration of immune cells through chemotaxis by secreting some cytokines and chemokines.

**Supplementary Information:**

The online version contains supplementary material available at 10.1186/s13045-022-01385-2.

**To the Editor,**

Recently, we reported the crystal structure of human CD47 in a complex with engineered SIRPα.D1 (N80A) [1]. The CD47 surface was buried by IMM01, a novel established new generation recombinant SIRPα-Fc fusion protein targeting CD47. Here, we report the anti-tumor therapeutic potential of IMM01.

IMM01 can significantly block the binding of a chimeric SIRPα receptor expressing cell (Jurkat-CSR) to CD47 (Additional file 1: Fig. S1A, B, C), inhibits the apoptosis of Jurkat-CSR cells via CD47-Fc (Additional file 1: Fig. S1D) and induces antibody-dependent cellular phagocytosis (ADCP) with an EC50 of 0.1389 nM (Additional file 1: Fig. S2A). The maximum antibody-dependent cell-mediated cytotoxicity (ADCC) levels were 1 nM and 5 nM for rituximab and IMM01 (Additional file 1: Fig. S2B), respectively. Rituximab induced strong complement-dependent cytotoxicity (CDC) activity in a dose saturation manner. IMM01 and Herceptin, however, showed no CDC induction activity (Additional file 1: Fig. S2C), implying that IMM01 induces strong ADCP and ADCC but not the CDC.

IMM01 has strong binding activity on all seventeen cancer cell lines, including Raji, Daudi, SU-DHL-10, Jurkat, HL60, MV-4-11, Reh, HCC827, NCI-H1299, NCI-H1975, A549, BT474, SK-BR-3, SK-OV-3, Hela, AGS and HT-29 (Additional file 1: Fig. S3), limited binding activities on human T, B, NK, and monocyte cells (Additional file 1: Fig. S4), and importantly, no binding activity on red blood cells (RBC). This indicates that IMM01 has a superior safety profile and will not have the so-called antigen sink phenomenon [2–6]. IMM01 reacts with cynomolgus CD47 but not with mouse or rat CD47 (Additional file 1: Fig. S5). IMM01 does not bind to human RBCs (Additional file 1: Fig. S6) to induce hemagglutination (Additional file 1: Fig. S7) and phagocytosis, but it binds to cynomolgus RBCs and induces phagocytosis (Additional file 1: Fig. S7). The N-linked glycosylation of CD47 protein contributes to the RBC non-binding attributes of IMM01 (Additional file 1: Fig. S8). Furthermore, IMM01 stimulates IL-10 and TNF secretion but not IL-1β, IL-2, IL-4, IL-5, IL-6, GM-CSF, and IFN-γ (Additional file 1: Fig. S9), indicating that IMM01 will not cause the cytokine release storm [7, 8].

IMM01 induced strong phagocytosis by binding to FcγRIIA and FcγRIIIA (Additional file 1: Fig. S10A, B), while IMM01M (D265A mutant) diminished phagocytosis due to the reduced binding activity to FcγRs. This suggests that the blocking axis of the CD47/SIRPα pathway not only needs to block the “don’t eat me” signal from CD47/SIRPα interaction but also needs to activate the “eat me” signal by the effective engagement of Fc with activating FcγRs in macrophages[2, 4–6].

The HL-60 xenograft model (Additional file 1: Fig. S11) demonstrated that 100% of the mice achieved complete remission (CR) after administration of IMM01, whereas 0% of the mice achieved CR after administration of IMM01M-inactive Fc for 2 weeks. Furthermore, 100% of the mice achieved CR after administration of IMM01 with the macrophages intact, whereas 0% of the mice achieved CR after macrophage depletion, indicating that IMM01 performs the therapeutic function through effective Fc function. The Daudi xenograft tumor model (Fig. 1A,B) revealed that the tumor growth inhibition (TGI) value was 97.48% on the day of 24 after administration of IMM01. A Raji orthotopic model showed that IMM01 has better effects than rituximab, and the combination of IMM01 and rituximab has significantly better results than any single drug alone (Fig. 1C).

**Fig. 1:**
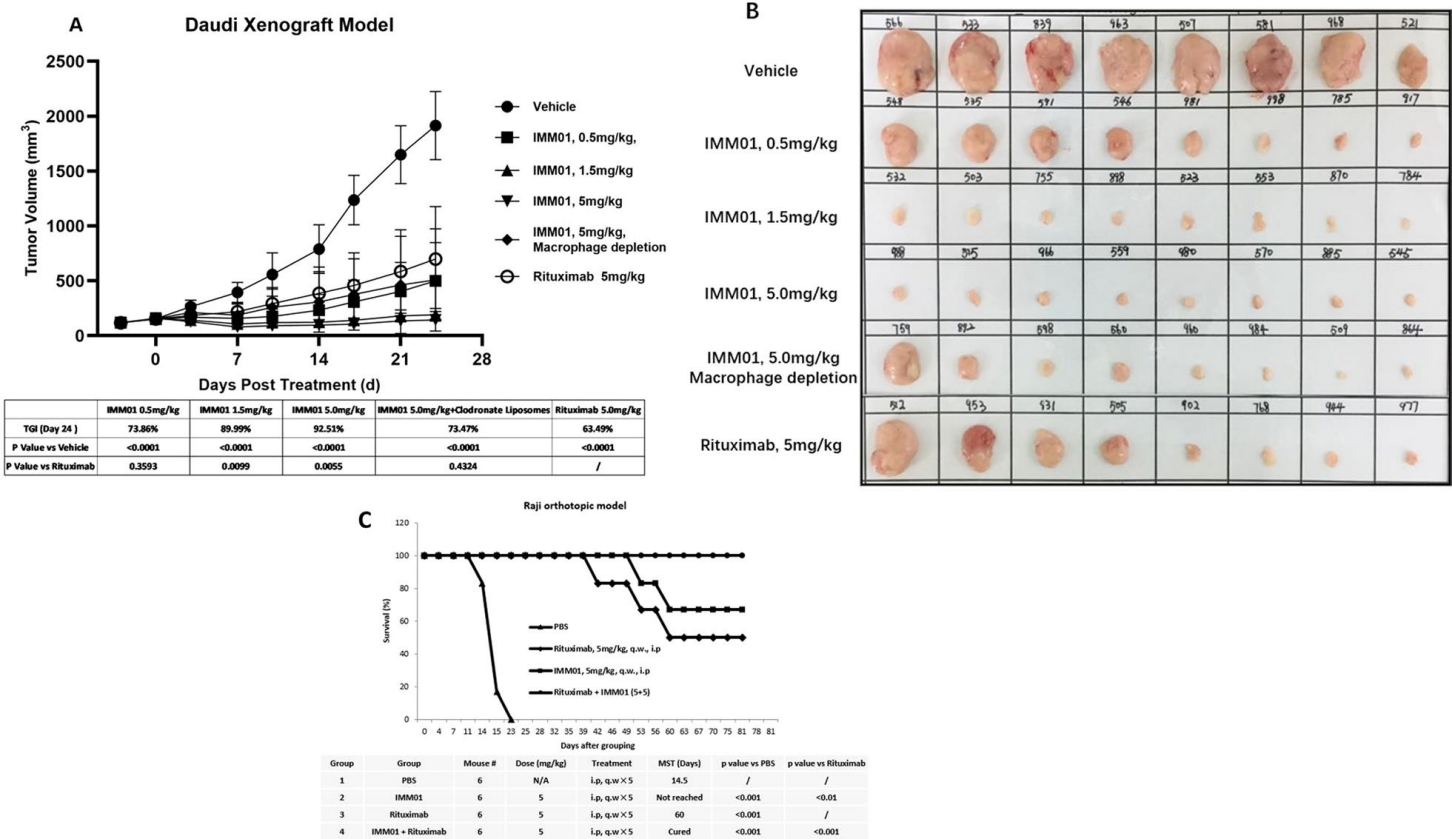
IMM01 anti-tumor activity against blood tumors was tested in the Daudi xenograft model (**A**, **B**) and the Raji orthotopic model (**C**). The Daudi xenograft tumor model (**A**, **B**) revealed that the tumor growth inhibition (TGI) value was 97.48% on the day of 24, after administration of IMM01 at 5 mg/kg. A Raji orthotopic model showed that IMM01 has better effects than rituximab, and the combination of IMM01 and rituximab (both at 5 mg/kg) has significantly better results than any single drug alone (**C**). IMM01 alone can prolong > 60% of the mice’s survival for 80 days, whereas the combination of IMM01 and rituximab can prolong 100% of the mice’s survival for 80 days

In CT26-hPDL1/hCD47 syngeneic tumor model in hPD-1/hSIRPα Tg Balb/c nude mice, the combination of IMM01 with PD-1 or PD-L1 antibody resulted in significantly stronger antitumor effects than PD-1 or PD-L1 alone (Fig. 2A,B). The efficacy of IMM01 alone and in combination with CD33 mAb and HER-2 mAb in the SNU-1 and HL-60 xenograft models (Fig. 2C, D), respectively, revealed great therapeutic potential in combination with these mAbs [9–12], as well as synergistic efficacy with pomalidomide and dexamethasone (Additional file 1: Fig. S12).

**Fig. 2:**
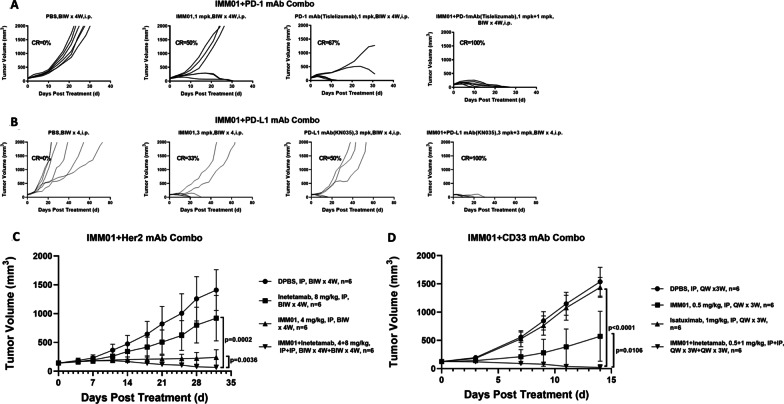
The IMM01 efficacy in combination with PD-L1 mAb (KN035) (**A**) and PD-L1 mAb (Tislelizumab) (**B**) on the in-house developed CT26-hPDL1(Tg) hCD47(Tg)mPDL1(KO)mCD47(KO) cell mouse tumor models, with CD33 mAb (Gemtuzumab) on HL-60 xenograft tumor model (**C**) and Her2 mAb (Inetetamab) on SNU-1 xenograft tumor model (**D**). It demonstrated that the combination of IMM01 with PD-1 or PD-L1 antibody resulted in significantly stronger antitumor effects than PD-1 or PD-L1 alone (**A**, **B**). The efficacy of IMM01 alone and in combination with CD33 mAb and HER-2 mAb in the SNU-1 and HL-60 xenograft models (**C**, **D**), respectively, revealed great therapeutic potential in combination with these mAbs

In summary, IMM01 exhibits strong anti-tumor activities with dual anti-tumor activities by blocking the CD47 “don’t eat me” signal and activating the phagocytosis “eat me” signal (Additional file 1: Fig. S13). It has good synergistic effects with different immunotherapeutic agents and has no human RBC binding activity and no hemagglutination induction. IMM01 inhibits anti-tumor activity via three possible mechanisms (Additional file 1: Fig. S14): (1) directly activating macrophages to phagocytize tumor cells; (2) presenting tumor antigens through MHC molecules to T cells; (3) activated macrophages can increase the infiltration of immune cells through chemotaxis by secreting some cytokines and chemokines. A phase I/II clinical trial of IMM01 combined with azacytidine for acute myeloid leukemia and myelodysplastic syndrome has been initiated (NCT05140811).

## Supplementary Information

**Additional file 1. **Supplement figures (Figs. S1–S15), materials and methods.

## Data Availability

The datasets analyzed during the current study are not publicly available.

## Abbreviations

ADCC: Antibody-dependent cell-mediated cytotoxicity
ADCP: Antibody-dependent cellular phagocytosis
CDC: Complement-dependent cytotoxicity
CR: Complete remission
mAb: Monoclonal antibody
RBC: Red blood cells
TGI: Tumor growth inhibition value
