# Supplementary material for: SIRPα-Fc fusion protein IMM01 exhibits dual anti-tumor activities by targeting CD47/SIRPα signal pathway via blocking the “don’t eat me” signal and activating the “eat me” signal
Source: J Hematol Oncol. 2022 Nov 16;15:167. doi: 10.1186/s13045-022-01385-2 (PMC9670587; doi:10.1186/s13045-022-01385-2)
Supplement: Supplementary file 1 — Additional file 1. Supplement figures (Figs. S1–S15), materials and methods. [file 13045_2022_1385_MOESM1_ESM.docx]

**Supplement file**

**Fig. S1.** Target binding on Jurkat cells and blocking activity on Jurkat-CSR cells.

Fig. S1A, S1B showed target binding on Jurkat cells; Fig. S1C shoed both SIRPα-Fc and IMM01 can significantly block the binding of CD47 to Jurkat-CSR cell. The IC50 of IMM01 was much lower than SIRPα-Fc, whereas the negative control rituximab showed no blocking activities. Fig. S1D. The CCK-8 method was used to measure Jurkat-CSR cell apoptosis induced by CD47-Fc blocked by IMM01 and TTI-621. The results revealed that IMM01 and TTI-621 can both significantly inhibit the apoptosis of Jurkat-CSR cells induced by CD47-Fc, and their blocking effects were similar. The CSR consists of the extracellular SIRPα domain sequentially connected by CD8a-hinge, CD28-TMD/ICD, and CD3ζ signal domain. When the Jurkat-CSR cells are incubated with recombinant CD47-Fc for 24-48 hours, the cells will undergo activation-induced cell death (AICD). However, when SIRPα-Fc was added, CD47-induced cell death was inhibited. With this method, IMM01 was compared with TTI-621 for the inhibiting activity of CD47-induced cell death, showing identical activity.

**Fig. S2.** IMM01 induced strong ADCC, moderate ADCC, and no CDC activity against Raji.

Fig. S2A. The ADCP assay showed that the IMM01 has an EC50 of 0.1399nM.

Fig. S2B. The ADCC assay on Raji cells showed that IMM01 has an EC50 of 0.8637nM.

Fig. S2C. CDC assay results showed rituximab can induce strong CDC activity in a dose saturation manner. However, IMM01 did not show any CDC induction activity.

**Fig. S3.** The IMM01 binding activity was accessed by flow cytometry on cancer cells. IMM01 has strong binding activity on all 17 cancer cell lines, including Raji, Daudi, SU-DHL-10, Jurkat, HL60, MV-4-11, Reh, HCC827, NCI-H1299, NCI-H1975, A549, BT474, SK-BR-3, SK-OV-3, Hela, AGS and HT-29, especially on Jurkat and HCC827 cells.

**Fig. S4.** The IMM01 binding activity was accessed by flow cytometry on normal blood cells. IMM01 has limited binding activity on human T, B, NK, and monocyte cells, and most importantly, no binding activity on RBC, nor hemagglutination induction, while the anti-human CD47 antibody hB6H12 showed strong binding activity on RBC as expected.

**Fig. S5.** IMM01 binding to different species’ CD47 was accessed by ELISA assay. IMM01 only reacts with cynomolgus CD47, not with mouse or rat CD47.

**Fig. S6.** IMM01 binding to human and cynomolgus, and phagocytosis against RBCs were detected by flow cytometry assay. IMM01 does not bind to human RBCs or induce phagocytosis against RBCs, but it can bind to cynomolgus RBCs and induce phagocytosis.


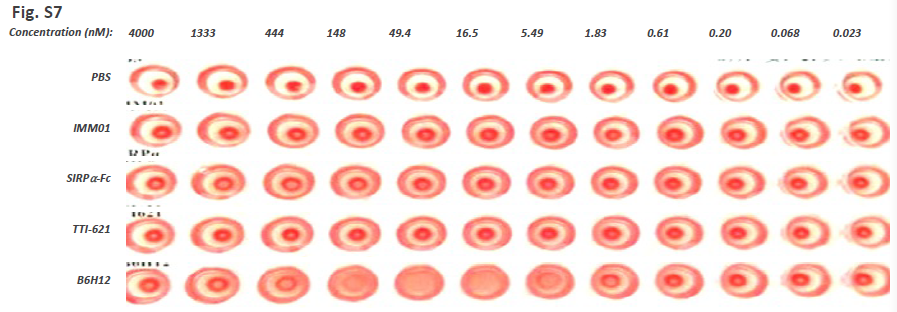


**Fig. S7.** IMM01 hemagglutination induction on human RBCs was accessed by a standard erythrocyte agglutination assay procedure. IMM01 has a favorable safety profile, with no human RBC binding activity and no hemagglutination induction.

**Fig. S8.** IMM01 binding to deglycosylated human RBCs was detected by flow cytometry assay. The results show that N-linked glycosylation of CD47 protein contributes to the RBC non-binding attributes of IMM01.

**Fig. S9.** Cytokine release was measured by cytometric bead array assay. IMM01 does not stimulate the secretion of cytokines of IL-1β, IL-2, IL-4, IL-5, IL-6, GM-CSF, and IFN-γ, but stimulates a significant amount of IL-10 and TNF production by PBMC from 12 donors. IMM01 significantly inhibited IL-8 secretion, which is about 40% of that treated with hIgG1-Fc (at a concentration above 1μg/mL). IMM0306 is a bispecific antibody targeting CD47 and CD20.


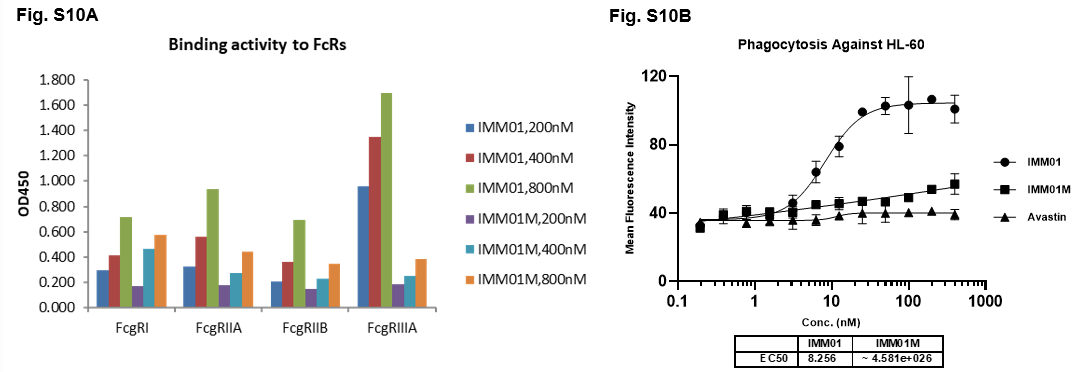


**Fig. S10.** IMM01/IMM01M binding to FcγRs and inducing phagocytosis were detected by flow cytometry. IMM01 binds to FcγRIIA and FcγRIIIA with high activity, while IMM01M (D265A mutant) significantly reduces the binding activity to FcγRIIA/FcγRIIB/FcγRIIIA. IMM01 significantly enhanced phagocytosis, while IMM01M dramatically diminished phagocytosis activity due to the reduced binding activity to FcγRs, especially FcγRIIA and FcγRIIIA.

**Fig. S11.** The HL-60 xenograft model demonstrated that 100% of the mice achieved complete remission (CR) after administration of IMM01 at 5 mg/kg for 2 weeks, whereas 0% of the mice achieved CR after administration of IMM01M-inactive Fc at 5 mg/kg for 2 weeks. This clearly indicates that IMM01 exerts therapeutic function by relying on effective Fc function.

**Fig. S12.** The efficacy of IMM01 in combination with Pomalidomide was evaluated in the NOD-SCID mouse subcutaneous xenograft model of MM tumor NCI-H929. The combination of IMM01 with pomalidomide plus/minus dexamethasone demonstrated excellent synergistic efficacy, indicating that it has great potential in combination therapy.


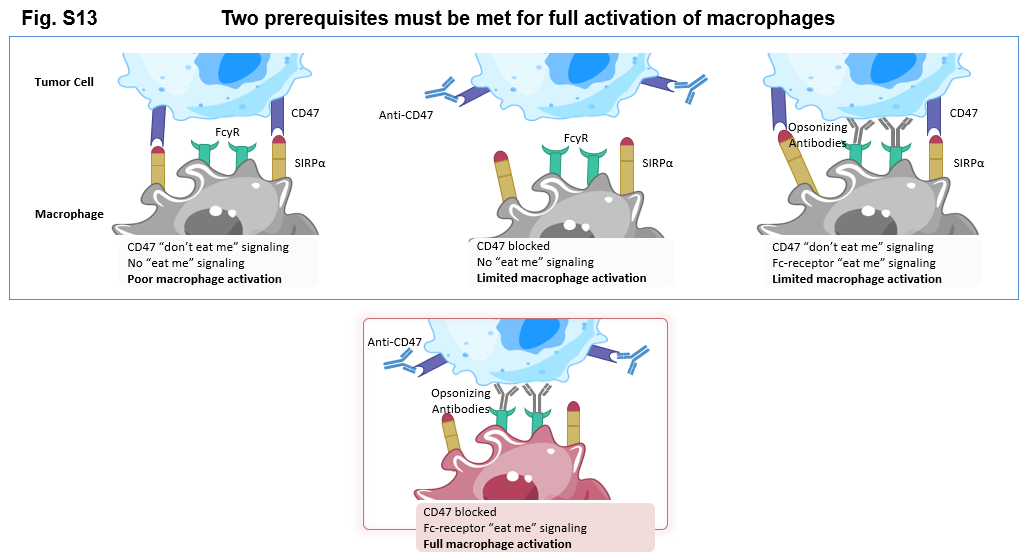


**Fig. S13.** Prerequisites for macrophage activation: IMM01 has strong dual anti-tumor activities by blocking the CD47 "don’t eat me" signal and activating the phagocytosis "eat me" signal, demonstrating good curative effects in mouse xenograft tumor models.


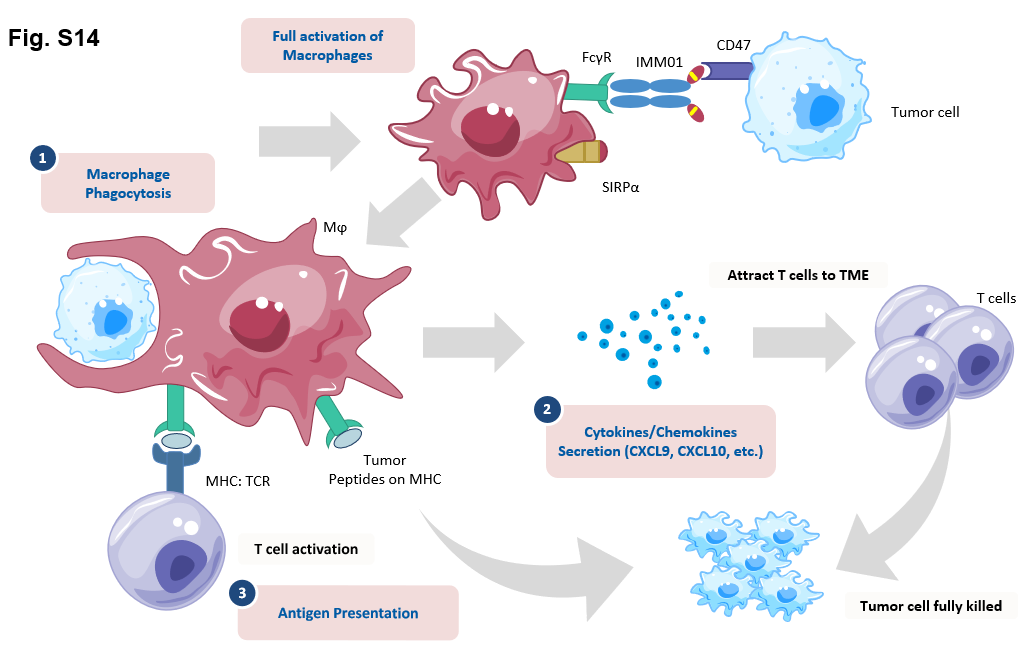


**Fig. S14.** Mechanism of action of IMM01: IMM01 inhibits the growth of tumor cells by the following three possible mechanisms: 1) directly activating macrophages to phagocytize tumor cells; 2) activated macrophages degrade phagocytized tumor cells and present tumor antigens through MHC molecules to activate T cells; 3) activated macrophages can convert "cold tumors" into "hot tumors" and increase the infiltration of immune cells through chemotaxis by secreting some cytokines and chemokines.

**Materials and methods**

1. **Animals, cell lines and healthy donors**

Severe combined immune-deficiency (SCID) CB17-SCID mice, C57/BL mice, BALB/c mice, were purchased from Beijing Vital River Laboratory Animal Technology Co., Ltd. BALB/c-hPD1SIRPα transgenic mice were provided by GemPharmatech Co. Ltd. All animal procedure were performed in compliance with Institutional Animal Care and Use Committee (IACUC) guidelines. In vivo anti-tumor response was evaluated using cancer cell line-derived xenograft. To avoid biological variables, only female mice were used for in vivo experiments. Treatment was initiated after tumors were established, average tumor volume of 100 mm^3^ when measured using m900 scanner (Piera, Brussels, BE). Before treatment, mice with small tumors (<50mm^3^) or infection signs were excluded from the experiments. The eligible mice were randomly assigned to each group. Tumor growth curves and overall survival were analyzed. The overall survival was defined as the time from start of treatment to when tumor volume reached 3000mm^3^ or mice death. To define the well-being of mice, changes in body weight, behaviors and physical appearance were monitored. All animal experiments were repeated at least 3 times to ensure the results were reliable. Detail procedures were described in the supplementary file.

PC-3 (Cat# TCHu158), Raw 264.7 (Cat# TCM13), Raji (Cat# TCHu44), Daudi (Cat# TCHu140), Jurkat (Cat# TCHu123), Reh (Cat# TCHu131), HCC827 (Cat# TCHu153), NCI-H1299 (Cat# TCHu160), A549 (Cat# TCHu150), BT-474 (Cat# TCHu143), SK-BR-3 (Cat# TCHu225), SK-OV-3 (Cat# TCHu185), Hela (Cat# TCHu187), Mv-4-11 (Cat # [SCSP-5031](https://www.cellbank.org.cn/search-detail.php?id=677)), NCI-H1975 (Cat # TCHu193), HT-29 (Cat # [TCHu103](https://www.cellbank.org.cn/search-detail.php?id=181)), HL-60 (Cat # [TCHu 23](https://www.cellbank.org.cn/search-detail.php?id=109)) and AGS (Cat # TCHu232) were purchased from the cell bank of the Chinese Academy of Sciences; SU-DHL-10 (Cat# CM-2045) was purchased from Shanghai Mingjin Biotechnology Co., Ltd. All cell lines were cultured under standard conditions. PC-3 cell were culture with Coons Modified Ham’s F12 + 2mM Glutamine + 7% Fetal Bovine Serum (FBS). HL60 cells were cultured in Iscove's modified Dulbecco's medium (IMDM; Gibco, Thermo Fisher Scientific) supplemented with 20% fetal bovine serum (FBS) (HyClone) and 1% penicillin/streptomycin solution (Gibco, Life Technologies) in an incubator at 37°C in the presence of 5% CO2. Raw264.7 cells were cultured at DMEM + 2mM Glutamine + 10% FBS. Jurkat, Raw 264.7 and Raji cells were cultured with advanced RPMI 1640 (GIBCO # 21870) + 10% fetal bovine serum (FBS; HyClone) + 2mM L-Glutamine + 100 units/ml penicillin + 100 mg/ml streptomycin (GIBCO #15140-122) + 10 mM Herpes with 5% CO2 at 37^o^C.

Blood samples were collected from healthy donors with written consent.

1. **Construction and expression of recombinant protein IMM01**

SIRPα-Fc is the recombinant fusion protein of SIRPα (V2) extracellular segment and human IgG1 Fc. SIRPαD1-Fc is the recombinant fusion protein of SIRPα (V2) extracellular segment domain 1 (D1) and human IgG1 Fc. IMM01 is the recombinant fusion protein of SIRPα (V2) extracellular segment domain 1 and human IgG1 Fc, with the N glycosylation site N80 mutated to A (Alanine) in D1 region. IMM01M contains a D265A mutation in IMM01 Fc segment, which eliminates or reduces the binding activity with Fc gamma receptors (FcγRs) **(as Fig. S15 below)**.


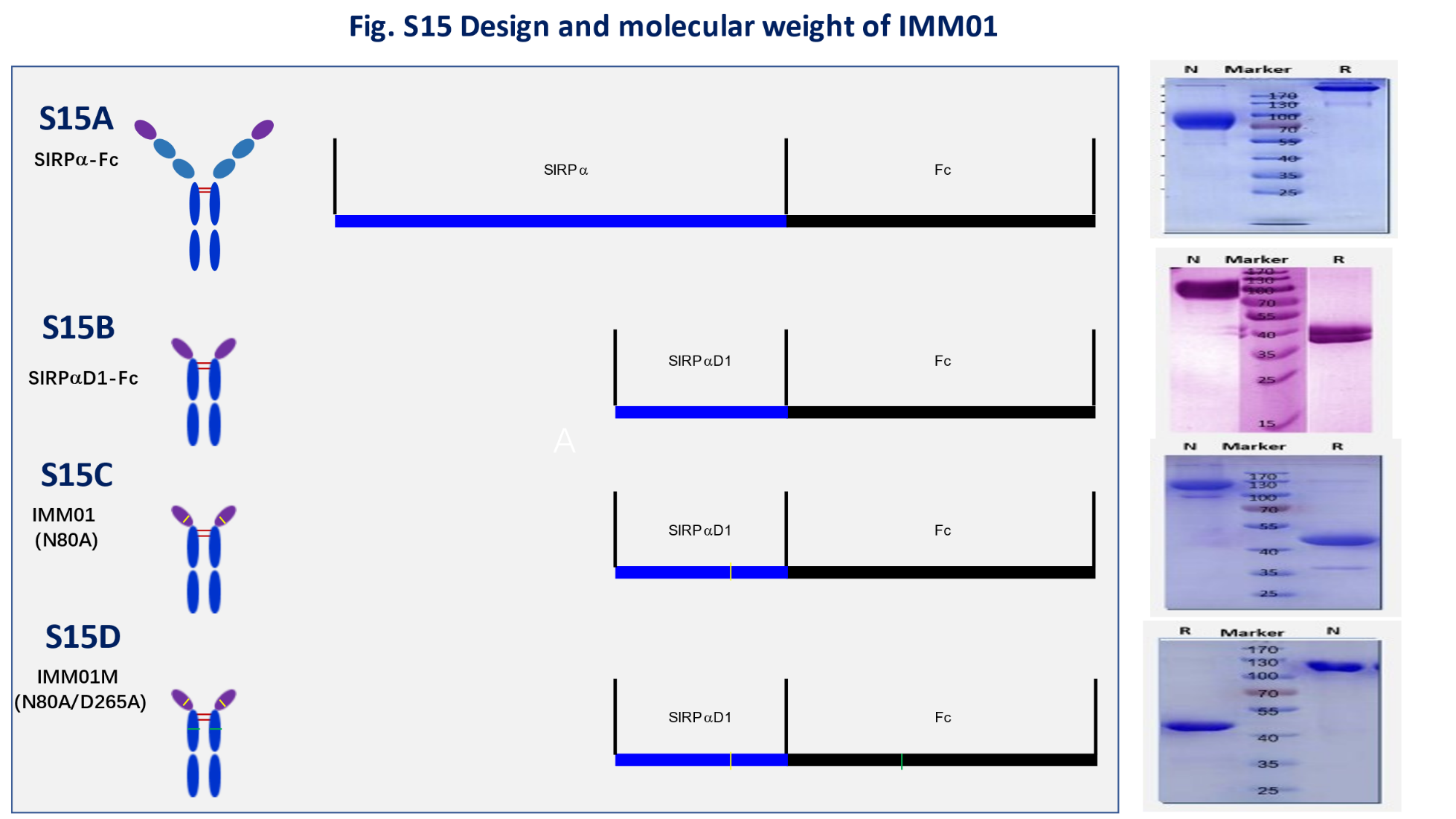
The nucleotide sequence encoding each fusion protein was synthesized by GenScript and was subcloned into a mammalian expression vector. SIRPα-Fc/SIRPα-D1-Fc/IMM01/IMM01M fusion proteins were produced using in house developed CHO-K1 cell expression system (ATCC# CCL-61) separately. Fusion proteins were purified with protein A affinity column chromatography. The purity of these fusion proteins was evaluated by size-exclusion high-performance liquid chromatography (SE-HPLC).

IMM01 recombinant fusion protein has not yet been commercialized. However, the fusion protein is being tested in a phase I clinical trial to evaluate the safety and tolerability of IMM01 combined with azacitidine in patients with AML and MDS and to explore the maximum tolerated dose (MTD) of IMM01 combined with azacitidine and determine the clinical recommended phase 2 dose (RP2D) of IMM01 combined with azacitidine (NCT05140811). Fusion protein IMM01 was generated by the method described previously in the patent of invention (CN106146670A) by ImmuneOnco Biopharmaceuticals (Shanghai) Co., Ltd.

1. **Antibodies and flow cytometry**

Commercial antibodies were obtained from different manufacturers as stated in the supplementary material (Table S1) unless otherwise stated. Flow cytometry was performed using the Guava EasyCyte 8HT with the GuavaSoft software. As a measure of antibody binding, the median fluorescence intensity (MFI) ratio was calculated by dividing the MFI of the tested antibody by the MFI of the corresponding isotypes. Antibodies were considered to bind the cells if the intensity exceeded an MFI ratio of 1.5.

1. **IMM01 blocking activity on Jurkat-CSR (Jurkat-CD47KO-CARSIRPα) cells**

CD47-Fc fusion protein (Sino Cat# 12283-HCCH), SIRPα-Fc and IMM01 proteins were diluted to 40nM with 1% BSA-PBS. 50ul of diluted protein solution was added into each well of 96-well U plates, respectively, starting from the highest concentration of 10000nM, following with 3-folds sequential dilution. The plates were incubated at 4**°C** for 45 min after mixing. A special cell line named as Jurkat-CSR modified from Jurkat cell line with human SIRPα overexpression and human CD47 knockout was established in-house by ImmuneOnco Biopharceuticals with the standard mouse gene overexpression and gene knockout procedures. Cultured Jurkat-CSR cells were collected after centrifugation at 1000RPM for 5 min and washed once with PBS solution. Cells were adjusted at the concentration of 1x10^6^/ml. 100ul of cell suspension was added into each well. The plates were then incubated at 4**°C** for 45 min after mixing. The plates were washed twice with 300ul of 1% BSA-PBS solution after incubation. Anti-human IgG (Fc)-FITC antibody（Sigma Cat# F9512）was diluted at 1:200 with 1% BSA-PBS solution and 100ul of antibody solution was added into each well of 96-well U plates. The plates were then incubated at 4**°C** for 45 min after mixing. The plates were washed once with 300ul of 1% BSA-PBS solution after incubation. Added 200ul of 1% BSA-PBS solution into each well. After vertex, samples were collected and analyzed by flow cytometry.

1. **Target affinity assay by Biacore**

HBS-EP+ was used as the experimental buffer, and each cycle included capture ligand, analyte injection and regeneration. With 10μL/min flow rate, 2.5 μg/ml antibodies were captured in 4 channels with anti-human Fc antibody for 50 seconds. The analytes were measured at the concentrations of 0 nM, 1.5625 nm, 3.125 nm, 6.25nm, 12.5nm, 25nm and 50nm, respectively. The analytes were injected into 3 and 4 channels of the chip at the speed of 30 μl/min flow rate. The binding time was set for 120 seconds, and the dissociation time was set for 120 seconds. Finally, glycine with pH 1.5 was used to regenerate the chip. The regeneration time was 30 seconds, and the flow rate was 30 μl/min. The temperature of the instrument was set at 25 °C.

1. **Antibody-dependent cellular phagocytosis (ADCP) ADCP assay**

Monocyte isolation and macrophage differentiation were used the standard method as described in supplementary file. Phagocytosis ratio was calculated with the formula: Phagocytosis (%) = experimental group phagocytosis (%)– blank control group phagocytosis (%).

Phagocytosis against tumor cells was done by using the method was used as described in the supplementary file. Ana-1 and HL-60 cells were used in the experiments and analyzed by flow cytometry to detect the green fluorescence signal of Ana-1 cells.

1. **Antibody-dependent cell-mediated cytotoxicity (ADCC) assay**

Raji cells were labeled with carboxyfluorescein succinimidyl ester (CSFE) and incubated with FcγRIIIA (158V) target-activated NK (FcR-TANK™) cells (developed in-house) at 1:2 ratio at 37 ℃ with 5% CO2 for 4 hours. Using Propidium Iodide (PI) solution to stain the cells before flow cytometry method was used to collect the cells and the PI positive staining cells were calculated. Calculation of ADCC intensity: Lysis% = (sample% PI positive cell - no antibody% PI positive cell) / (100 - no antibody% PI positive cell) x 100%.

1. **Complement-dependent cytotoxicity (CDC) assay**

Raji cells were incubated with different concentrations of IMM01, and standard rabbit complex complement at 37 ℃ with 5% CO2 for 4 hours, followed by staining with PI solution. The flow cytometry method was used to collect the cells and the PI positive staining cells were calculated. Calculation of CDC intensity was done by using the following formula: Lysis %= Experimental Sample Lysis %- No Antibody Lysis %.

1. **Detection of Jurkat-CSR cell apoptosis induced by CD47-Fc blocked by IMM01 and TTI-621**

Jurkat-CSR cell apoptosis induced by CD47-Fc blocked by IMM01 and TTI-621 was measured by CCK-8 method. Detailed method was described in supplementary file.

1. **Cross-reaction with different species’ CD47**

Two different types of standard sandwich ELISA assays were used to measure the cross-reaction with different species’ CD47 by coating IMM01 on the plates and coating the CD47 on the plates, respectively. The absorbance values were read at 450nM. The results were analyzed by GraphPad Prism 8.0 ^®^ software with four parameters.

1. **Binding Activity of IMM01 and CD47 positive tumor cells**

Different cell lines were cultured under standard conditions. Cells were stained with PE anti human CD47 (Biolegend, cat#323108) or PE mouse IgG1 (Biolegend, cat# 400114), incubated for 45min in a 4°C followed by flow cytometry analysis. For binding test, TTI-621, IMM01, HB6H12 and hIgG1-Fc were diluted to different concentrations and then incubated with cells followed by staining with secondary antibody anti-human IgG (Fc) – FITC. Then the cells were analyzed flow cytometry to measure the binding ability of IMM01 to different tumor cells. The data were further analyzed with GraphPad Prism 8.0 software. The relative MFI=（MFI test – MFI secondary antibody）/ MFI unstained.

1. **Binding activity to normal blood cells** **of different lymphocyte subtypes**

Isolated peripheral mononuclear cells (PBMC) and platelets were incubated with antibodies TTI-621, IMM01, HB6H12 and hIgG1-Fc at 4°C for 45min. Then the cells were stained with fluorescence conjugated antibodies with PE anti-human CD14 (Biolegend, cat # 367104), PE anti human CD20 (Biolegend, cat #302306), PerCP anti-human CD3 (BD Biosciences, cat#552851), and PE/Cy5 anti-human CD56 (Biolegend, cat#318308), followed by flow cytometry analysis.

1. **Cytokine release assay by CBA**

After treatment of the PBMC with different conditions, supernatants were collected, and cytokines were measured by using BD CBA assay kits. The Data processing and analysis were done by using BD CBA FCAP array software. The procedures were used with the standard process according to the manufacturer recommended protocol. Detailed method was described in the supplementary file.

1. ***In vivo*** **mechanism of action study in mouse models**

Human leukemia cell HL-60 CB17-SCID, Daudi CB17-SCID and human gastric cancer SNU-1 cells CB17-SCID mice xenograft subcutaneous tumor models were established by cell inoculation method: tumor cells were collected in logarithmic growth stage and subcutaneously inoculated into the SCID mice with 2x10^6^/0.1ml /mouse cells suspension in 1xPBS with Matrigel at 1:1 ratio at the cell concentration to 2x10^7^/ml. When the tumor volumes reached 100-200 mm^3^, the animals were randomly divided into different groups with the tumor difference between each group was less than 10% of the mean value. Different agents were administered to the different group mice. Tumor volumes and the body weight of the animals were measured three times a week, and the clinical symptoms were observed and recorded once a day.

CB17-SCID mice xenograft orthotropic Raji tumor model was established by inoculating 0.2ml cell suspension at the concentration of 5x10^6^/mouse by caudal vein. Three days after inoculation, animals were randomly divided into different groups with the body weight difference between each group was less than 10% of the mean value. Starting on day 0, the drugs were administered according to the weight of animals. During the administration, if the weight of individual animals decreased by more than 15% compared to day 0 (BWL≧15%), the drug will be stopped until the weight of animals recovered (BWL≦15%). During the experiment, the body weight of the animals was measured three times a week, and the clinical symptoms were observed and recorded once a day.

Mouse colon cancer cell CT26-hPDL1(Tg) hCD47 (Tg) mPDL1 (KO) mCD47 (KO) mouse tumor models were also established with the standard allogeneic transgenic procedure. tumor cells in logarithmic growth stage and resuspend it in RMPI-1640 medium at 2x10^7^/ml. Tumor cells were subcutaneously inoculated into the right back of BALB/c-hPD1SIRPa transgenic mice at 2x10^6^/0.1ml/mouse.

When the tumor volume reached 100-120 mm^3^, the animals are randomly divided into different groups with the tumor difference between each group was less than 10% of the mean value. IMM01 was administered side by side with different agent combination with Rituximab, KN035, tislelizumab and inetetamab as the controls. Tumor volumes and the body weight of the animals were measured three times a week, and the clinical symptoms were observed and recorded once a day.

1. **Human red blood cells (RBCs) binding assay**

Human RBCs were diluted and incubated with TTI-621, IMM01, hIgG1-Fc and HB6H12 at different concentrations, followed by staining with secondary antibody anti-human IgG (Fc)-FITC (Sigma, cat# F9512). Cells were analyzed by flow cytometry to measure the binding activity of IMM01. A total of 100 normal donors (62 males and 38 females) were tested by flow cytometry. The binding activity of IMM01 to human erythrocytes before and after glycosidase treatment was compared by flow cytometry.

1. **Erythrocyte agglutination test**

To evaluate the erythrocyte agglutination ability of SIRPα-Fc fusion protein IMM01, Human erythrocyte agglutination assay was done with the standard test procedure. Human erythrocytes were diluted to 1% in PBS. Different concentrations of TTI-621, IMM01, HB6H12 and hIgG1-Fc were incubated in the round bottom 96-well plates at room temperature for 1-2 hours. The presence of non-precipitated RBCs indicates signs of hemagglutination, which is foggy compared to the punctate red spots of non-hemagglutinating RBCs.

1. **Statistical analysis.**

SPSS and Graphpad Prism 8.0 software (San Diego, CA, USA) are used for statistical analysis, and the results are expressed as mean ± standard deviation. Statistically significant differences between different groups were analyzed using Student t-test. Kaplan Meier survival curves were used to compare survival rates between different groups. *P* < 0.05 indicates a statistically significant difference.
